# Supplementary material for: Complexome profiling of the Chlamydomonas psb28 mutant reveals TEF5 as an early PSII assembly factor
Source: Plant Cell. 2025 Mar 17;37(6):koaf055. doi: 10.1093/plcell/koaf055 (PMC12164590; doi:10.1093/plcell/koaf055)
Supplement: koaf055_Supplementary_Data [file koaf055_supplementary_data.zip › psb28_interactive.zip!/plots/intensity/qualityPlots/peptides/_Cre01.g043450.t1.2.html]

### Cre01.g043450.t1.2

Trivial name:   
SeqCoverage [median]: 39.20  
Mass: 22.29 kDa  
  
 

| Sequence | MissedCleavage | Mass [Da] | wtIntensity | muIntensity | Score |
| --- | --- | --- | --- | --- | --- |
| AAAQIESR | 0 | 844.44 | 4606.43 | 9229.92 | 109.01 |
| AAQDATDDVNVVLDK | 0 | 1572.76 | 0.00 | 1546.19 | 118.76 |
| ANALAAEK | 0 | 786.42 | 0.00 | 4872.13 | 116.73 |
| ANTAQLEEK | 0 | 1002.50 | 1998.35 | 3247.59 | 182.98 |
| AVEAQVTSVENQLQQK | 0 | 1770.91 | 0.00 | 4021.60 | 251.24 |
| KAVEAQVTSVENQLQQK | 1 | 1899.01 | 0.00 | 8338.27 | 220.34 |
| LDRVEMLQK | 1 | 1130.61 | 1007.60 | 9122.18 | 92.05 |
| QQLEYILAQQEIAR | 0 | 1701.90 | 0.00 | 1843.36 | 57.41 |
|  |  |  | 7612.4 | 42221.2 |  |
